# Establishment of a novel mouse model of colorectal cancer by orthotopic transplantation

**DOI:** 10.1186/s12885-025-13834-5

**Published:** 2025-03-06

**Authors:** Cewen Chen, Qiaochu Fu, Lei Wang, Shinya Tanaka, Masamichi Imajo

**Affiliations:** 1 Department of Cancer Pathology, Faculty of Medicine, Hokkaido University, N15, W7 Kita-Ku, Sapporo, 060-8638 Japan; 2 Institute for Chemical Reaction Design and Discovery (WPI-ICReDD), Hokkaido University, N21, W10 Kita-Ku, Sapporo, 001-0021 Japan

**Keywords:** Colorectal cancer, Orthotopic model, Cecal epithelium, Transplantation

## Abstract

**Background:**

Colorectal cancer (CRC) represents a major malignancy that poses a significant threat to human health worldwide. The establishment of a reliable and pathologically relevant orthotopic model of CRC is crucial for gaining a deeper understanding of its molecular mechanisms and for developing more effective therapies. Nonetheless, the development of such models is fraught with challenges primarily owing to the technical complexities associated with the transplantation of CRC cells into the intestinal epithelium.

**Methods:**

The luminal surface of the cecum was externalized to visualize the entire process involved in the transplantation of CRC cells into the cecal epithelium of BALB/c athymic nude mice. The cecal epithelium was mechanically removed, preserving the integrity of the submucosal layer. Caco-2 CRC cells were subsequently inoculated onto the epithelium-depleted surface of the cecum to reproduce the development of CRC within the epithelial layer. The successful removal of the epithelium and transplantation of Caco-2 cells were verified through the use of appropriate fluorescent labeling techniques and examination with a fluorescence stereoscopic microscope.

**Results:**

Following orthotopic transplantation, Caco-2 cells formed tumors in the cecum, where tumors progressed from a flat monolayer epithelium to thickened aberrant crypt foci, and then to protruding polyps, aided by mesenchymal cells infiltrating the tumors to form a stalk region, and eventually to large tumors invading the submucosa. Throughout this process, Caco-2 cells retained stem cell and fetal intestinal signatures, regardless of their location within the tumors or their proliferative status. Histopathological analysis further suggested that interactions between the transplanted Caco-2 cells and the surrounding normal epithelial and mesenchymal cells play critical roles in tumor development and in the elimination of normal epithelial cells from the tumor in this model.

**Conclusions:**

This study established a novel orthotopic model of CRC within the mouse cecum. Tumor development and progression in this model include sequential morphological changes from a flat monolayer to large invasive tumors. The establishment of this orthotopic CRC model, which mimics tumor development in a more natural microenvironment, provides new opportunities to investigate the molecular mechanisms underlying CRC and to evaluate novel anticancer therapies in pathologically relevant contexts.

**Supplementary Information:**

The online version contains supplementary material available at 10.1186/s12885-025-13834-5.

## Introduction

Colorectal cancer (CRC) is a major malignancy that poses a global threat to human health [1, 2]. Among all cancers, it ranks third in incidence and cancer-related deaths [2]. Consequently, there is an increasing demand for the development of novel therapeutic options that can effectively improve patient outcomes. The establishment of a suitable in vivo model of CRC not only helps us to better understand the molecular mechanisms underlying CRC initiation and progression but also provides a bridge between preclinical research and clinical trials for the development of new anticancer drugs [3, 4]. To date, subcutaneous xenograft models have been widely used in cancer research because of their convenience in transplanting cancer cells and monitoring tumor growth [5, 6]. Nonetheless, subcutaneous tumor models cannot provide an appropriate cancer microenvironment resembling the original organs/tissues, limiting the translatability of the obtained results to actual human cancers [3, 7]. Indeed, subcutaneous cancer models often lack complex interactions between cancer cells and the microenvironment, which are essential for analyzing cancer growth and responses to therapy in the correct tissue context [3, 7]. Therefore, orthotopic cancer models that more accurately replicate clinically relevant biological contexts are needed for a better understanding of cancer development and the advancement of new therapies. Over the past two decades, several orthotopic mouse models of CRC have been proposed. In most of these models, however, CRC cells are inoculated outside or between the muscularis externa, resulting in tumor development within a microenvironment that is distinct from the original epithelium [8–12]. Owing to the technical difficulties associated with the transplantation of cancer cells into the intestinal epithelium, a reliable orthotopic transplantation model for CRC has yet to be established. As a result, it remains challenging to recapitulate the interactions between CRC cells and the natural environment resembling the original tumors. Considering that the tumor microenvironment exerts significant effects on tumor cell identity and behavior [13, 14], the establishment of suitable animal models of CRC would be valuable for elucidating the mechanisms of CRC development and developing novel therapeutic strategies.

In this study, we aimed to establish an orthotopic transplantation model for CRC that could replicate cancer development in the natural tissue microenvironment. Given that the interactions between CRC cells and adjacent normal epithelial cells, submucosal stromal cells, and immune cells play critical roles in both tumor suppression and growth [7, 15–20], we postulated that the intestinal epithelium represents the most appropriate site for the inoculation of CRC cells. To establish a protocol for transplanting CRC cells into the intestinal epithelium, we developed a surgical technique that allows direct observation and access to the luminal surface of the cecum. Under the guidance of a stereoscopic microscope, all procedures for surgical transplantation of CRC cells into the cecal epithelium were successfully visualized, leading to efficient engraftment of the transplanted cells and subsequent initiation of tumors. Tumors originated as small foci surrounded by normal epithelial cells and later expanded into larger, homogeneous tumors while suppressing the proliferation of adjacent normal epithelial cells. Invasive foci were also observed in proximity to the primary tumors. In all types of tumor foci observed, the majority of CRC cells maintained their original features, such as proliferative, undifferentiated phenotypes and similar expression profiles of pathological markers. Our results establish a novel surgical methodology for inducing CRC development within the mouse intestinal epithelium.

## Methods

### Cell culture

Caco-2 human colon cancer cells and HEK293T cells were obtained from the American Type Culture Collection and maintained in Dulbecco's modified Eagle’s medium (DMEM) supplemented with 10% fetal bovine serum (FBS), 2 mM glutamine, and antibiotics (100 U/ml of penicillin). Cells were grown at 37℃ in a humidified atmosphere with 5% CO_2_. To establish a Caco-2 cell line stably expressing a fluorescent marker, we used a lentiviral vector encoding mScarlet. For the production of lentivirus, HEK293T cells were transfected with the pCSII-mScarlet-IRES-puro plasmid, together with the psPAX2 and pCMV-VSV-G-RSV-Rev plasmids. The culture supernatants containing the lentiviruses were harvested 48 h after transfection, filtered, concentrated, and subsequently employed to infect Caco-2 cells. Following infection, puromycin selection was performed to eliminate noninfected cells. For titration of the lentiviruses, Caco-2 cells were seeded at a density of 50,000 cells/well in a 12 well plate, and then infected with mScarlet-expressing lentiviruses at serial dilutions. Two days after infection, the percentage of mScarlet-positive cells was quantified. A sample showing 10–20% infection efficiency was used to calculate the lentiviral titer (transduction unit).

### Orthotopic xenograft model of colorectal cancer in mice

Female BALB/c athymic nude mice (8–12 weeks old) were purchased from CLEA Japan, Inc., and subjected to transplantation of Caco-2 cells into the cecal epithelium. Prior to surgical intervention, the mice were anesthetized with isoflurane and placed in a supine position. The abdomen was disinfected with alcohol and a small longitudinal incision (1.5–2.0 cm) was made in the abdomen. The cecum was exteriorized through this incision, and a small longitudinal incision was made at the distal edge of the cecum. The luminal contents of the cecum were expelled using cotton swabs, after which the cecum was inverted from the incision to expose its luminal surface. The cecum surface was stained with 10 ~ 20 μl of Fast Green FCF solution for several minutes to visualize the epithelial structures. The Fast green dye has been employed in protein staining in biochemical assays [21] and for highlighting tissue architecture by staining the extracellular matrix [22]. When used to stain the cecum surface, it was found to be useful for delineating the contours of tissues, such as the location of crypts, by staining the cell surface proteins. The cecum surface was then brushed to remove epithelial cells. The efficacy of this epithelial cell removal method was confirmed by visualizing cell nuclei with Nuclear Green LCS1 (AAT Bioquest, #17,540), which was diluted 1:250 in phosphate-buffered saline (PBS) and directly applied to the cecal surface. After 5 min incubation, fluorescent signals from Nuclear Green LCS1 were observed to confirm the absence of epithelial cells within the crypts using a fluorescent stereoscopic microscope. Caco-2 cells expressing mScarlet were expanded under standard culture conditions, detached by trypsinization, and suspended in PBS immediately before inoculation. An 8 μl cell suspension containing 1 × 10^5^ cells was applied to the surface of the cecum and allowed to adhere for ten minutes. The cecum was subsequently repositioned into its original orientation, sutured and reintroduced into the peritoneal cavity. The abdominal wall and skin layer were sutured with a 6–0 nylon suture. The mice were monitored until they regained consciousness and mobility. The tumors were allowed to grow for up to 16 weeks. In total, ten mice were analyzed in this study: two were examined within a week, three after one month, and five beyond three months post-transplantation. In all cases, successful engraftment of the transplanted Caco-2 cells was observed. In addition to the tumor xenograft experiments, two mice were sacrificed immediately after the removal of the epithelium to analyze the tissue status. Apart from these cases, during the method’s development, two mice died unexpectedly, probably due to post-operative bleeding. However, once the procedure was refined, no further unexpected deaths occurred. Therefore, although this orthotopic CRC model requires technical proficiency, the tumor formation rate was considered high. To observe mScarlet fluorescence in living animals, the mouse cecum surface, on which Caco-2-mScarlet cells were seeded, was observed with a SZX16 fluorescence stereomicroscope (Olympus) equipped with a DP74 high-resolution microscope digital camera, filter units containing excitation and emission filters (green fluorescence: BP460-495 and BA510IF (SZX2-FGFP), red fluorescence: BP530-550 and BA575IF (SZX2-FRFP1)), and SDFPLAPO0.5XPF and SDFPLAPO1XPF objective lenses. The X-Cite LED-based system was used as an excitation light source. Images were taken by the microscope under the control of the cellSens software and then analyzed using the Image J software. After euthanasia of mice, the developed tumors were excised and fixed with 10% neutral buffered formalin. This study was approved by the institutional animal care and use committee of Hokkaido University.

### Histology and immunofluorescence

Xenograft specimens were washed with cold PBS, and fixed overnight in 10% formalin at 4℃. For histopathological evaluation, hematoxylin and eosin (H&E) staining was performed on paraffin-embedded xenograft sections. The following antibodies were used for immunofluorescence: RFP (Proteintech, 5F8), Ki-67 (Abcam, ab15580), CD133 (Cell Signaling Technology, D2V8Q), E-cadherin (Cell Signaling Technology, 4A2), TROP2 (TACSTD2) (Abcam, ab214488), Vimentin (Cell Signaling Technology, D21H3), COL1A1 (Cell Signaling Technology, E8F4L), and ATAD2 (Cell Signaling Technology, E8Y7F). For immunofluorescent staining, the xenograft sections were treated with 0.5% Triton X-100 in Tris-buffered saline (TBS) for 10 min. Antigen retrieval was performed by treating the samples at 98℃ for 20 min in 10 mM citrate buffer, pH 6.0. After blocking with 1% bovine serum albumin (BSA) in TBS containing 0.05% tween-20, the sections were incubated with primary antibodies diluted in the blocking buffer overnight at 4℃. Alexa Fluor 488 conjugated goat anti-rabbit IgG (Molecular Probes, no. A-11008), Alexa Fluor 594 conjugated goat anti-rabbit IgG (Molecular Probes, no. A-11037), Alexa Fluor 594 conjugated goat anti-mouse IgG (Molecular Probes, no. A-11032), Alexa Fluor 488 conjugated goat anti-mouse IgG (Molecular Probes, no. A-11029), and fluorescein–conjugated goat anti-rat IgG (Proteintech, SA00003-11) were used as secondary antibodies. Nuclei were stained with Hoechst33342 (Dojindo). For EdU staining, mice were injected intraperitoneally with EdU (80 μg/g body weight) 6 h before euthanasia. EdU staining was performed by treating the sections with PBS containing 4 mM CuSO_4_, 10 μM fluorescein-azide, and 100 mM ascorbic acid for 30 min. For quantification of UEA-I-positive goblet cells, the total numbers of the epithelial cell nuclei and those of cells containing the UEA-I-positive secretory granules in five different regions of the control normal mucosa and the tumor-adjacent mucosa were counted using Image J software. Statistical comparison was performed using the unpaired Student’s t test. A *p* value less than 0.05 was considered statistically significant.

### Expression analysis of fetal intestinal signature genes in CRC cell lines

The expression of fetal intestinal signature genes, such as *CLU*, *LY6E*, and *TACSTD2*, in CRC cell lines was analyzed using data obtained from the DepMap Portal (https://depmap.org/portal) [23]. The data were analyzed utilizing the R statistical software.

## Results

### Microscope-guided removal of the cecal epithelium

**Fig. 1:**
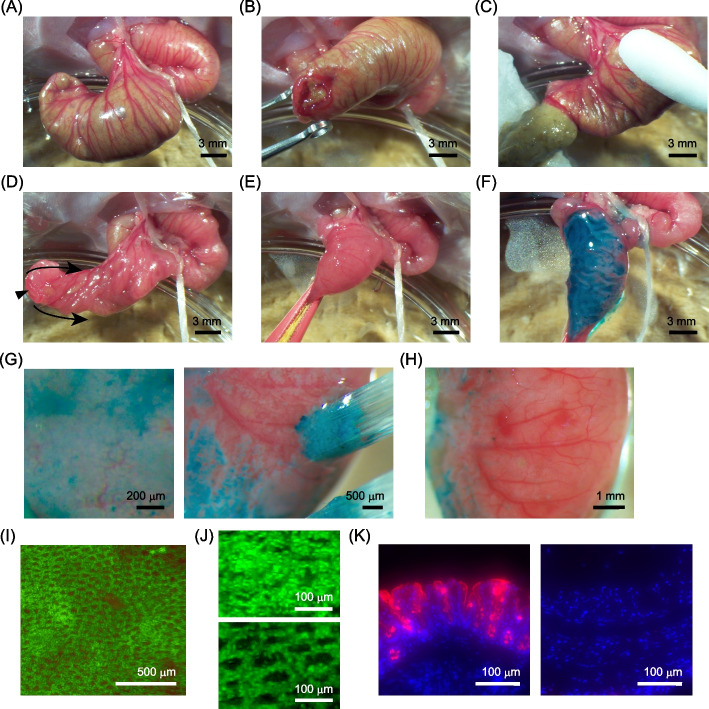
Establishment of surgical procedures for the externalization and removal of the cecal epithelium. **A** Gross view of the mouse cecum. **B** A small incision was made at the distal end of the cecum. **C** The luminal contents of the cecum were pushed out of the incision. **D** After the removal of the luminal contents, the cecal wall was inverted from the incision to expose the luminal epithelium. **E** Gross view of the externalized luminal surface of the cecum. **F** The cecal surface was stained with Fast Green to visualize the epithelial layer. **G** A magnified view of the cecal surface stained with Fast Green. Individual crypts were identifiable as small white spots, which were efficiently removed by mechanical brushing. **H** Gross view of the epithelium-removed surface of the cecum. **I**, **J** Confirmation of the absence of epithelial cells within the crypts via Nuclear Green staining. Each black hole represents a single crypt devoid of epithelial cells. **J** Magnified view of the cecum surface before and after epithelial removal. **K** Staining of mucus granules with Rhodamine-UEA-I (red) present in the differentiated epithelial cells of the cecum. Nuclei were stained with Hoechst33342 (blue). Comparison of sections from the normal (left) and epithelium-removed (right) cecum confirmed the successful removal of the differentiated cells

CRCs arise from the colorectal epithelium as a result of the sequential accumulation of genetic and epigenetic alterations [1, 24, 25]. These alterations confer niche independency on cancer cells, resulting in their massive outgrowth from the epithelial stem cell compartment [24, 25]. To replicate tumor development within the epithelium, we aimed to develop a surgical approach for transplanting CRC cells into the intestinal epithelium of immunodeficient mice. For this purpose, we first exteriorized the luminal surface of the mouse cecum by making a small incision at its edge and then inverting the cecum through this incision (Fig. 1A-E). The luminal surface of the cecum was then stained with Fast Green FCF dye to facilitate the visualization of epithelial structures (Fig. 1F). The staining with Fast Green enabled us to distinguish the presence and absence of epithelial cells within each crypt (Fig. 1G). To expose the extracellular matrix layer that serves as a scaffold for epithelial cell adhesion, the cecal epithelium was mechanically removed by brushing the cecum surface under observation with a stereoscopic microscope (Fig. 1G). The successful removal of the epithelium was validated by the appearance of empty crypt holes deprived of cells inside (Fig. 1H). To further validate the absence of epithelial cells, we employed the fluorescent dye, Nuclear Green, to stain the cell nuclei. The nuclei were initially distributed across the entire surface of the tissue, but after the removal of epithelial cells, a region devoid of nuclei appeared in the central region of each crypt (Fig. 1I, J), suggesting that the epithelial cells within the crypts were removed, whereas stromal cells surrounding the crypts were still preserved. Finally, sections of the cecum were examined to assess the tissue condition immediately after the removal of the epithelium. Immunofluorescent staining showed that most, but not all, epithelial cells were successfully removed by brushing, while the underlying collagen layer remained (Supplementary Fig. S1A). The staining of cell nuclei and differentiated epithelial cells also revealed that the majority of differentiated cells, but not the underlying stromal cells located in the submucosal layer, were removed by these procedures (Fig. 1K).

During epithelial removal, we occasionally observed minor bleeding from the cecal surface (Supplementary Fig. S1B). However, the bleeding was not severe and could be halted by applying pressure with a cotton swab. The reason why we experienced only minor bleeding could be attributed to the preservation of the large blood vessels. In support of this, immunofluorescent staining of collagen also showed that the submucosal layer beneath the epithelial layer remained relatively intact (Supplementary Fig. S1A). Collectively, these results demonstrate the effective removal of the cecal epithelium and conservation of the intact submucosal layer.

### Transplantation of CRC cells into the cecal epithelium

**Fig. 2:**
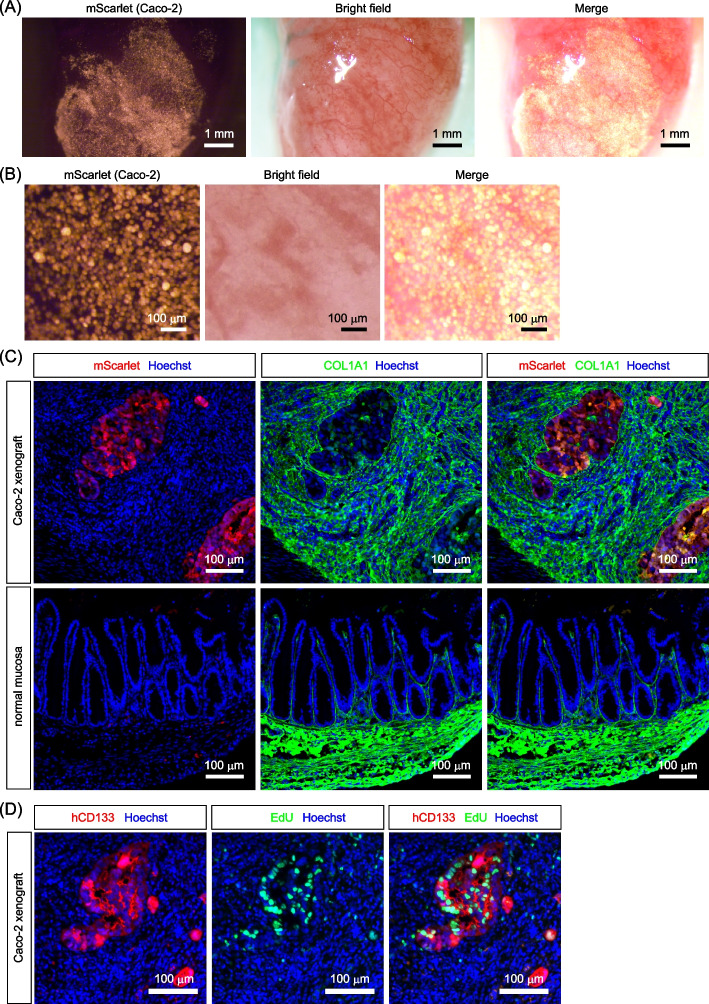
Transplantation of Caco-2 colorectal cancer cells into the cecal epithelium. **A** Caco-2 cells expressing the fluorescent marker, mScarlet, (Caco-2-mScarlet) were seeded onto the epithelium-removed surface of the cecum. **B** A magnified view of the cecal surface upon the seeding of Caco-2-mScarlet cells. **C**, **D** Immunofluorescence analysis of the cecum one week after the transplantation of Caco-2-mScarlet cells. **C** mScarlet-positive Caco-2 cells formed many tumor foci within the cecum, which were surrounded by the dense collagen layer. **D** Proliferating cells were stained by EdU labeling. As it has been demonstrated that CD133 is expressed at the detectable levels in most Caco-2 cells [28, 29], an antibody specific for human CD133 was used to label Caco-2 cells. Many transplanted Caco-2 cells exhibited positivity for EdU

We next examined whether CRC cells could be efficiently engrafted onto the epithelium-depleted surface of the cecum. For transplantation into the cecal epithelium, we selected Caco-2 cells, a well-known CRC cell line with relatively well-differentiated features. For identification after transplantation, Caco-2 cells stably expressing the red fluorescent protein mScarlet were generated using a lentivirus vector (Supplementary Fig. S2A-C). After selection with the antibiotic puromycin for more than two weeks, mScarlet expression could be maintained in Caco-2 cells cultured in the absence of puromycin for at least two weeks (Supplementary Fig. S2C), suggesting that mScarlet expression was relatively stable in this cell line. Caco-2-mScarlet cells were directly seeded onto the epithelium-depleted surface of the cecum and incubated for ten minutes (Fig. 2A, B). The cecum was then reoriented, sutured, and returned to the peritoneal cavity. These simple procedures achieved efficient engraftment of Caco-2 cells into the cecum. One week after transplantation, many Caco-2 xenograft foci had formed (Fig. 2C). Following epithelial removal, the cecum undergoes rapid regeneration, during which the surface of the wound bed containing an increased number of stromal cells is covered with stem cells supplied from adjacent crypts [26, 27]. Consistent with these studies, one week after transplantation, we observed the formation of a thickened wound bed containing abundant stromal cells, which seemingly encapsulated Caco-2 cells (Supplementary Fig. S3A). Thus, although Caco-2 cells were initially placed on the surface of cecum and present at the location corresponding to the original epithelial layer, the thickened wound bed seems to cover these regions thereafter. The abundance of stromal cells in the wound bed might lead to collagen production, as COL1A1 staining exhibited higher intensity in this region (Fig. 2C). mScarlet fluorescence was clearly observed in the transplanted tissues, but not in the untransplanted normal tissues or epithelium-depleted tissues (Supplementary Fig. S3B). We then examined the proliferation status of the engrafted Caco-2 cells by labeling proliferating cells with a thymidine analogue, 5-ethynyl-2’-deoxyuridine (EdU). EdU staining revealed the presence of many proliferating cells in the tumor foci (Fig. 2D), suggesting that Caco-2 cells began to proliferate soon after engraftment into the cecal epithelium. Thus, our methodology has enabled efficient transplantation of CRC cells into the cecal epithelium.

### Development of various types of tumors from transplanted Caco-2 cells

**Fig. 3:**
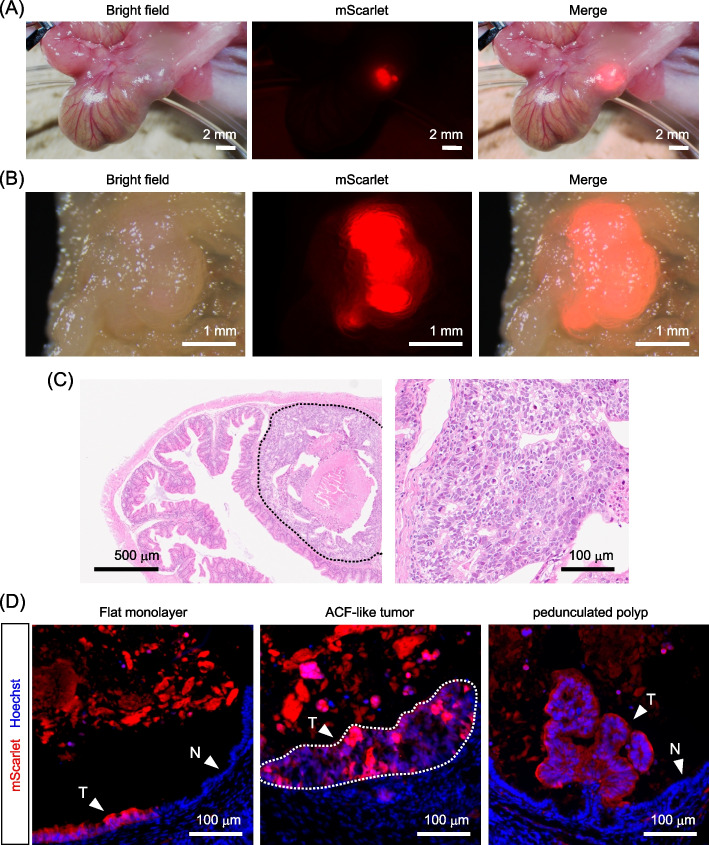
Development of various types of small tumors by transplanted Caco-2-mScarlet cells. **A** Gross view of the cecum one month after the transplantation of Caco-2-mScarlet cells. Small tumor foci expressing mScarlet were visible from the outside of the cecum. **B** Caco-2-mScarlet cell tumors observed from the luminal side after dissection. **C** H&E staining of the cecum one month after transplantation of Caco-2-mScarlet cells. Low- (left) and high-magnification (right) images are shown. The dotted line encircles the xenografted region. **D** Distribution of Caco-2-mScarlet (red) cells in different types of tumors. (left) In the flat monolayer tumor foci, Caco-2 cells (T) were present directly adjacent to the normal epithelial cells (N) that were negative for mScarlet. Caco-2 cells also formed ACF-like foci, which were exclusively composed of mScarlet-positive tumor cells (center), and protruded, pedunculated tumors supported internally by host stromal cells (right). These tumors (T) were also found in proximity to the normal epithelium (N)

The successful engraftment of Caco-2 cells into the cecal epithelium encouraged us to observe tumor development from the transplanted cells. One month after transplantation, the Caco-2 cells formed tumors of visible size under a stereoscopic microscope (Fig. 3A, B). Since the normal epithelial cells migrated and covered the surface of the wound bed, a novel epithelial layer formed above the regions where transplanted Caco-2 cells exist (Fig. 3C). Consequently, Caco-2 cell tumors often seemed to be located beneath the newly formed normal epithelial layer (in the submucosal layer). However, as Caco-2 cells were initially seeded on the surface of the cecum, at least in some sections, the lumen of the tumor regions was found to be connected to the intestinal lumen (Supplementary Fig. S4A), suggesting that Caco-2 cells were still present in the outermost epithelial layer. Tumors could be clearly distinguished from the adjacent normal epithelium by observing the fluorescence of mScarlet expressed by Caco-2 cells (Fig. 3A, B). No metastatic foci were observed outside the cecum. Morphologically, Caco-2 cells formed different types of tumors, including a simple flat monolayer, thickened, abnormal gland-like epithelium resembling aberrant crypt foci (ACF), and relatively large protruded, pedunculated polyp-like tumors (Fig. 3D). In these tumors, Caco-2 cells were present adjacent to normal epithelial cells, mimicking CRC growth within the surrounding normal epithelium (Fig. 3D). Since a small portion of epithelial cells remained after mechanical brushing (Supplementary Fig. S1A), these remaining cells, as well as the transplanted Caco-2 cells, should proliferate in the wound area, likely resulting in adjacency and competition between normal and tumor cells. In addition, in some cases, we observed large cellular masses containing epithelial glandular structures, as well as Caco-2 tumors (Supplementary Fig. S4B). These regions primarily consisted of mScarlet-negative and E-cadherin-positive epithelial cells (Supplementary Fig. S4B). We think that these regions were formed by normal epithelial cells remained after mechanical brushing (Supplementary Fig. S1A).

**Fig. 4:**
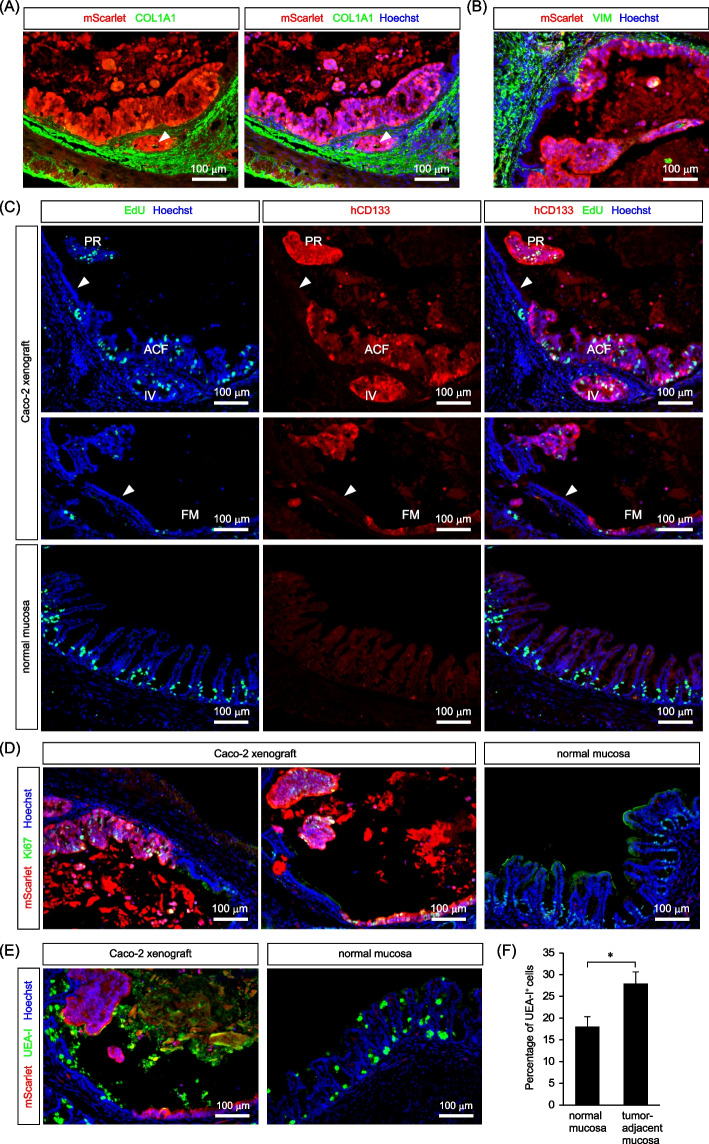
Immunofluorescence analysis of early-stage Caco-2 cell tumors. **A-E** Immunofluorescence analysis of various types of Caco-2 cell tumors one month after transplantation. **A** Tumor foci were predominantly located within the region corresponding to the original epithelial layer, with their basal side supported by a collagen layer. However, small invasive foci were occasionally observed in the submucosa beyond the collagen layer, as indicated by the white arrowhead. **B** Distribution of vimentin (VIM)-positive mesenchymal cells surrounding Caco-2 cell tumors. **C** Cell proliferation analysis in different types of Caco-2 cell tumors by EdU staining. Various types of mScarlet-positive Caco-2 cell tumors, such as flat monolayers (middle, FM), ACF-like (top, ACF), protruded (top, PR) and invasive tumors (top, IV), exhibited a significant presence of EdU-positive tumor cells. By contrast, normal epithelial cells adjacent to tumors were deficient in cell proliferation (top and middle, white arrowheads). (bottom) Control mouse cecum with many proliferating normal epithelial cells. **D** Positivity of Caco-2 tumors for the cell proliferation marker, Ki-67. Ki-67-positive cells were present abundantly in tumor regions, whereas adjacent normal epithelial cells were mostly quiescent. **E**, **F** Staining of differentiated goblet cell granules with rhodamine-UEA-I. Tumors were deficient in UEA-I-positive cells, while they were abundant in the adjacent normal epithelium. **F** Percentage of UEA-I-positive goblet cells in the normal mucosa and tumor-adjacent mucosa (mean ± SEM). *, *p* < 0.05 (Student’s t test)

One month after transplantation, the distribution of Caco-2 cells was mostly restricted within the region corresponding to the original epithelial layer, as evidenced by the presence of a dense collagen layer lining beneath the tumor cells (Fig. 4A). However, a small fraction of the cells invaded the submucosal layer beyond the collagen layer (Fig. 4A). These invasive foci were often observed near ACF-like tumors (Fig. 4A), suggesting the origin of the invasive foci. Many mesenchymal cells were observed in the submucosal layer beneath Caco-2 cells (Fig. 4B). The mesenchymal cell lining was also observed inside the protruded tumors, suggesting the recruitment of mesenchymal cells by transplanted Caco-2 cells (Fig. 4B). We then examined the cell proliferation status in each type of tumor foci by EdU staining. EdU-positive cells were evenly distributed in all types of tumor foci, including the flat monolayer, ACF-like, protruded, and invasive tumors, (Fig. 4C), demonstrating vigorous cell proliferation in these tumors. By contrast, normal epithelial cells adjacent to tumor foci were mostly negative for EdU (Fig. 4C), suggesting that Caco-2 cells suppressed the proliferation of adjacent normal epithelial cells. Similar results were obtained after staining with another cell proliferation marker, Ki-67 (Fig. 4D). We also examined the distribution of mucus-producing goblet cells, a subtype of differentiated cells in the cecum. The staining of mucus granules with a fluorescently labeled lectin protein (rhodamine-UEA-I) revealed that the goblet cell differentiation was promoted in the normal epithelium adjacent to the tumor foci, whereas Caco-2 cells were almost completely negative for the staining (Fig. 4E, F). These results suggest that Caco-2 cells maintain their proliferative, undifferentiated state during tumor development, while promoting the differentiation of adjacent normal epithelial cells.

### Development of large invasive tumors from transplanted Caco-2 cells

**Fig. 5:**
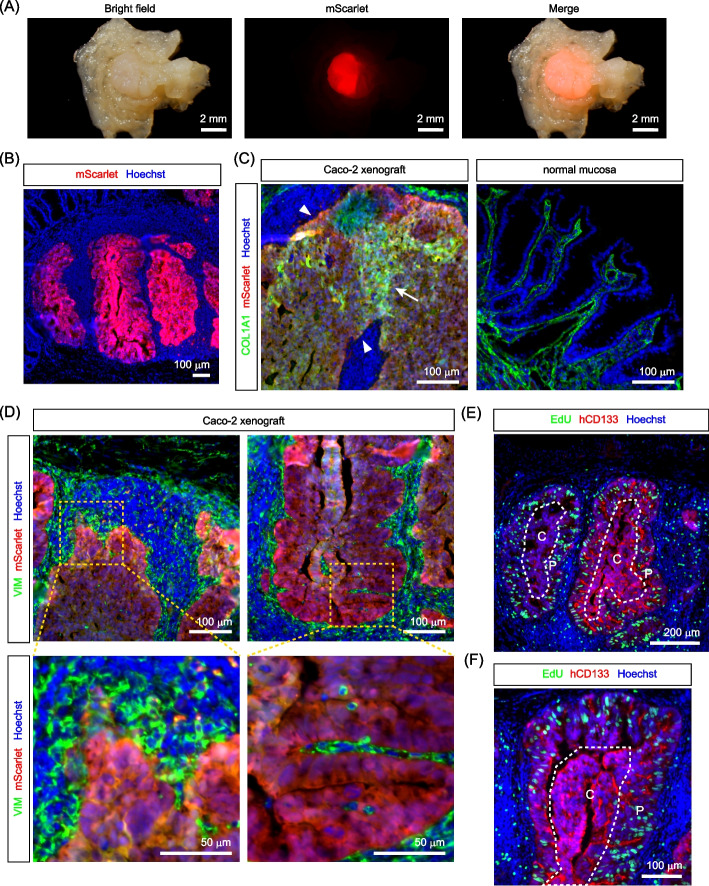
Development of large invasive tumors by transplanted Caco-2-mScarlet cells. **A**, **B** Caco-2-mScarlet cell tumors developed three months after transplantation. **A** Gross (top) and magnified (bottom) views of the Caco-2 cell tumor from the luminal side. **B** Caco-2 cells (red) formed large tumors, characterized by complex folding of the tumor epithelium. Nuclei were stained with Hoechst33342. **C**-**F** Immunofluorescence analysis of large invasive Caco-2 cell tumors. **C** The dense collagen layer observed at earlier stages was absent at the interface between the large invasive Caco-2 cell tumors and the surrounding stromal cells (indicated by white arrowheads). In these tumors, Caco-2 cells exhibited moderate positivity for COL1A1 (indicated by a white arrow), suggesting the expression of collagen in these cells. **D** Caco-2 cells within large invasive tumors directly interacted with vimentin (VIM)-positive mesenchymal cells. The vimentin-positive cells infiltrated the folds of the Caco-2 cell sheet (right). **E**, **F** Analysis of proliferating cells by EdU staining. In the large tumors, EdU-positive proliferating cells were predominantly located in the peripheral regions (P), with a notable absence in the central regions (C)

As mentioned above, at one month after transplantation, the tumors developed were relatively small and mostly localized in the region corresponding to the original epithelial layer, with the exception of small invasive foci. However, three months after transplantation, Caco-2 cells formed much larger tumors with complex epithelial folding inside (Fig. 5A, B). These large tumors comprised a homogenous Caco-2 cell population and were deficient in normal epithelial cells (Fig. 5B). Thus, normal epithelial cells present adjacent to tumor cells at the earlier stages had already been excluded, presumably because of the competition with tumor cells. Unlike the small tumors observed at the earlier stages, the large tumors were not necessarily encircled by the apparent collagen layer, which was present in some areas around the tumor, but disappeared from the other areas (Fig. 5C). In areas depleted of collagen, epithelial cells appeared to interact directly with vimentin-positive mesenchymal cells (Fig. 5D). Compared with small tumors at earlier stages, large tumors were surrounded by many more mesenchymal cells, some of which had infiltrated the inside of the tumors (Fig. 5D), suggesting active recruitment of mesenchymal cells by tumor cells. The presence of mesenchymal cells seemed to be significant for tumor growth, as EdU staining revealed that tumor cells located in the central region of these tumors, where mesenchymal cells had not yet infiltrated, were mostly negative for EdU, whereas the peripheral region adjacent to abundant mesenchymal cells contained many EdU-positive cells (Fig. 5E, F). Alternatively, the lower proliferation of tumor cells in the center of large tumors can also be attributed to hypoxia, the examination of which represents an important issue for future studies. These observations suggest that Caco-2 cells develop into large invasive tumors through recruiting and interacting with mesenchymal cells in the submucosal layer.

Tumor angiogenesis may play a significant role in CRC progression and infiltration. Thus, we investigated tumor angiogenesis by immunofluorescent staining of VEGFR2, which is specifically expressed in endothelial cells. We found that, one month after transplantation, VEGFR2-positive endothelial cells were scarce around the Caco-2 cell tumors; however, three months later, they were abundant around the tumors (Supplementary Fig. S5). Since Caco-2 cells were also positive for VEGFR2, we could not compare the number of endothelial cells inside the tumor mass. Thus, further investigation is needed to confirm angiogenesis in tumors and its role in tumor progression. Nevertheless, the increase in the number of endothelial cells around tumors suggests that tumor angiogenesis might occur vigorously during the progression to large invasive tumors.

### Investigation of intestinal stem cell and cell plasticity marker expression in Caco-2 cell tumors

**Fig. 6:**
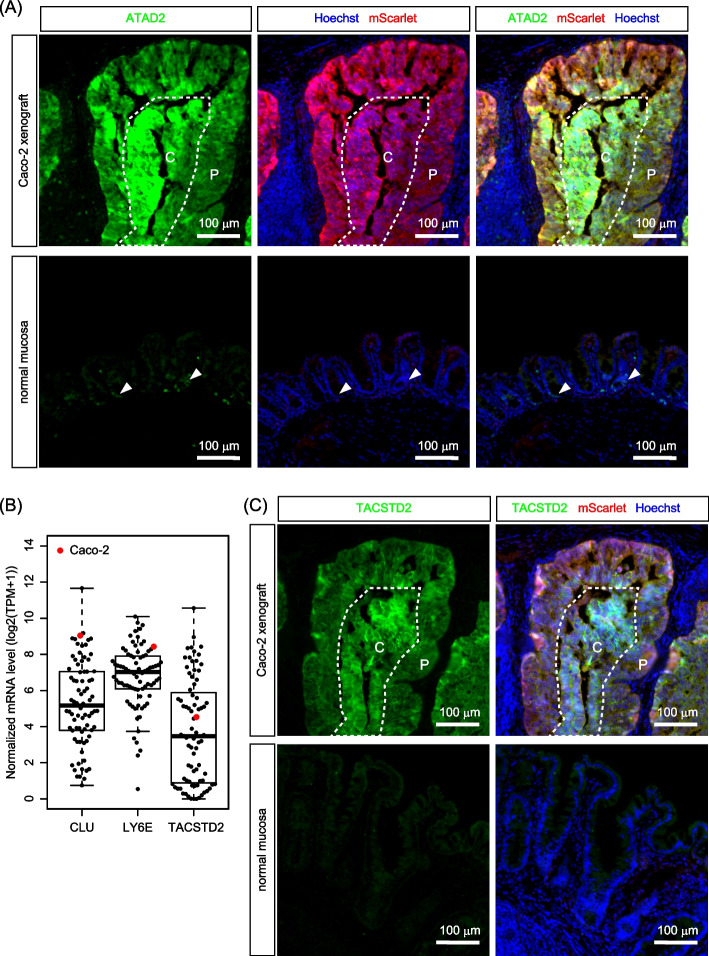
Investigation of intestinal stem cell and fetal intestinal marker expression in tumors derived from Caco-2 cells. **A** In Caco-2 cell tumors, an intestinal stem cell marker, ATAD2, was expressed in the majority of tumor cells. The expression levels of ATAD2 were similar in both the central (C) and peripheral (P) regions of large invasive tumors. By contrast, normal mucosa exhibited a significantly lower expression of ATAD2, which was restricted to epithelial cells located at the crypt base. **B** The mRNA expression levels of fetal intestinal signature genes across eighty CRC cell lines. **C** A fetal intestinal marker, TACSTD2, was expressed in the majority of tumor cells in Caco-2 tumors. TACSTD2 expression was comparable in both the central (C) and peripheral (P) regions of large invasive tumors. The normal cecal epithelium lacked TACSTD2 expression

We next investigated whether Caco-2 cells within tumors retained the characteristics of intestinal stem cells. To address this, we analyzed the expression of a recently identified intestinal stem cell marker, ATAD2 [30, 31], by immunofluorescence. In normal mucosa, ATAD2 was expressed exclusively in cells at the base of the crypts (Supplementary Fig. S6A), indicating the presence of intestinal stem cells in that area. In early-stage Caco-2 cell tumors, ATAD2 was ubiquitously expressed in most tumor cells (Supplementary Fig. S6B). This suggests that Caco-2 cells retain their intestinal stem cell characteristics during tumor development. By contrast, normal epithelial cells located adjacent to tumor cells lost ATAD2 expression (Supplementary Fig. S6B), supporting the notion that these cells had undergone differentiation and consequently lost their stem cell characteristics. In larger invasive tumors that developed subsequently, ATAD2 expression was still high in the majority of Caco-2 cells (Fig. 6A). Notably, while the central regions of these tumors were deficient in proliferating cells, ATAD2 expression was still maintained at high levels in these areas (Fig. 6A). This observation suggests that Caco-2 cells within the central regions of larger tumors become quiescent presumably due to a lack of supportive mesenchymal cells, yet they appear to retain stem cell-like phenotypes. This highlights a distinction in the requirements of the tumor microenvironment for the maintenance of cancer stemness versus cell proliferation. Notably, Caco-2 cells exhibited high ATAD2 expression under standard culture conditions (Supplementary Fig. S6C), showing that Caco-2 cells could maintain high ATAD2 expression in both in vitro culture and in vivo tumor environments.

Recent studies have shown that intestinal epithelial cells can be reprogrammed into the fetal intestine-like state during tissue regeneration and tumorigenesis [32–34]. Such plasticity likely plays a significant role in CRC development and progression. Indeed, in human CRCs, the expression of a fetal gene signature is associated with drug resistance, metastasis and poor prognosis [33, 35, 36]. Furthermore, in a mouse model of intestinal adenoma, spatiotemporal reprogramming that induces the fetal intestine-like state is constitutively activated, resulting in the emergence of heterogenous tumor cell populations [32]. Consistent with the documented reactivation of fetal gene signatures in CRCs, our analyses revealed that Caco-2 cells expressed fetal intestine-specific genes at relatively high levels among eighty CRC cell lines (Fig. 6B). Consequently, we examined whether Caco-2 cells within established tumors expressed fetal intestinal markers. The results indicated that a fetal intestinal marker, TACSTD2 (also known as TROP2), is highly expressed in Caco-2 cell tumors but not in the normal epithelium (Fig. 6C). TACSTD2 expression was comparable in both the proliferative (peripheral) and quiescent (central) regions of large tumors (Fig. 6C). These findings suggest that the expression of a fetal signature is not affected by the cell proliferation status of Caco-2 cell tumors. Taken together, these findings imply that Caco-2 cells maintain the expression of both intestinal stem cell and plasticity markers independently of their spatial location within the tumors.

## Discussion

The establishment of an orthotopic model that replicates the natural tumor microenvironment is crucial for advancing cancer biology and the development of novel therapeutic strategies; however, this remains a significant challenge for many types of cancers [3]. Since the submucosal layer of the mouse intestine is very thin, it is technically challenging to inject cells into this layer. Consequently, in CRC research, many studies have employed protocols that involve the inoculation of CRC cells into the cecal wall, specifically within or outside the muscularis externa [8–12]. Nevertheless, the microenvironment of these tissues markedly differs from that of the intestinal epithelium, where actual human CRC originates. For instance, CRC cells transplanted into these regions are deprived of critical interactions with normal epithelial cells [15, 16, 37], stromal cells including trophocytes and myofibroblasts [17, 19, 20], and immune cells [7, 18]. These interactions play a vital role in regulating the maintenance of stemness and differentiation within the intestinal epithelium. The absence of appropriate cell–cell interactions has constrained the clinicopathological relevance of previously developed CRC transplantation models, underscoring the necessity for the development of more accurate orthotopic transplantation models.

In this study, we have established a novel orthotopic model of CRC that mimics natural colorectal tumor development within the mouse cecum. The development of tumors in this model includes sequential morphological changes. Initially, the engraftment of transplanted Caco-2 CRC cells was confined to the region corresponding to the original epithelial layer (Fig. 2). One month after transplantation, Caco-2 cells formed several morphologically distinct types of tumors (Fig. 3). The emergence of multiple tumor types at the early stages of tumor development can be attributed to sequential progression from the flat monolayer epithelium to the thickened ACF-like epithelium, followed by the formation of protruded polyps with the help of mesenchymal cells infiltrating into tumors to establish a stalk region (Fig. 4). Then, three months after transplantation, the Caco-2 cell tumors progressed to large invasive tumors harboring heterogeneous cell proliferation states, influenced by variations in the microenvironment (Fig. 5). Therefore, the novel orthotopic CRC model established in this study provides a robust framework for inducing CRC development from flat monolayer tumors to large invasive tumors within the cecum. Given that Caco-2 cells originate from a mature CRC, they have already acquired sufficient mutations to form large invasive tumors. Thus, we think that the observed morphological changes result from complex interactions between tumor cells and the surrounding tissue environment. For instance, the formation of protruding polyps requires the recruitment of mesenchymal cells, which exist beneath the surface tumor cells within the protrusion. We also observed many endothelial cells surrounding large invasive tumors (Supplementary Fig. S5), which potentially promotes the development of the tumor vasculature to support the growth of large tumors. Since it takes time for tumor cells to recruit stromal cells and alter tissue architecture, tumors may initiate as a flat monolayer before undergoing sequential morphological changes to progress into large tumors. Therefore, we believe that the morphological alterations observed in our mouse model were caused by changes in tumor-microenvironment interactions and subsequent changes in tissue architecture, rather than the accumulation of novel genetic mutations.

The tumorigenicity of Caco-2 cells is relatively modest compared with that of other CRC cell lines. Thus, although several studies have used this cell line in xenograft experiments [38, 39], other more invasive CRC cell lines have been more prevalent in previous studies. Thus, the successful development of Caco-2 xenografts suggests the superior ability of our model as an orthotopic CRC model. Importantly, our results showed that Caco-2 cells began proliferation one week after transplantation (Fig. 2D). This suggests that the microenvironment of the cecum surface remaining after epithelial removal could sufficiently support the proliferation of the transplanted cells in this model. We think that preservation of such a favorable microenvironment is one of the advantages of this model.

In this study, the cecum was chosen as the site for CRC cell transplantation owing to its two advantages. First, in the cecum, it was relatively easy to make a small incision at its distal end and evert to expose its luminal surface (Fig. 1). In general, such invasive manipulation of the intestinal tract often leads to severe adhesions, resulting in fatal intestinal obstruction in mice. However, while adhesions and tissue deformation were observed in the cecum, no deaths due to intestinal obstruction occurred in this model. This allowed us to directly observe and manipulate the luminal surface of the intestine and to transplant cells into the epithelial layer. Thus, tolerance to invasive surgical procedures is an advantage of the cecum as a transplantation site in establishing orthotopic CRC models. Second, in this model, even when the tumors grew into protrusions on the luminal side, no signs of intestinal obstruction were observed. This allowed the tumors to grow sufficiently large within the mouse intestine, thereby enabling the replication of massive invasive tumors (Fig. 5). These findings indicate that the cecum is an excellent transplantation site for creating orthotopic colorectal cancer models because of its advantages such as surgical feasibility and resistance to intestinal obstruction. Importantly, exposure to fecal content could be deleterious to the transplanted cells in the cecum; therefore, the transplantation site was first isolated from fecal flow in a previous study reporting syngeneic transplantation of intestinal cells into the rat cecum [40]. As mice are much smaller than rats, invasive surgery, including massive resection, is generally difficult. However, as we removed the fecal content from the cecum before transplantation, the transplanted cells were likely prevented from contacting it until fecal flow brought new feces. As it took more than an hour to complete all procedures and wake the recipient mouse from anesthesia, it is assumed that the transplanted cells would not contact the fecal content for a few hours. We think that the time without contact with feces contributes to improving the survival rate of the transplanted cells, leading to their efficient engraftment into the tissue.

Previously, several studies have reported CRC models that implant tumor cells in the intestinal epithelium. In most models, mice are treated with dextran sulfate sodium (DSS) to induce colitis before transplantation [41, 42]. DSS administration causes topical injury to a fraction of the colon epithelium, creating an exposed site of the ECM to which CRC cells can adhere. However, the extent and location of the damage are probabilistic, which can lead to large variations in the engraftment rate of cancer cells. In addition, in most previous protocols, transplantation procedures are not visible; one cannot directly confirm the wound and seed the cells on it. In contrast, the surgical procedures developed in this study enabled the direct observation and creation of the wound and topical seeding of CRC cells on the wound. We believe that the visualization of the transplantation procedures significantly improved the engraftment rate of the transplanted cells and the reproducibility of the tumor models.

It should be noted that the cecum should undergo vigorous regeneration upon removal of the epithelium, which could induce changes in the tissue microenvironment. Indeed, intestinal epithelial injury is often accompanied by infection and inflammation, resulting in activation of immune systems [43]. The activation of immune cells, including macrophages and innate lymphoid cells, may affect the development and progression of tumors, as it has already been shown to affect epithelial cell functions during regeneration. Thus, elucidating the role of these immune cells in tumor development and progression would be the next important issue in future studies.

Tumor-stromal cell interactions play a crucial role in the development and progression of CRCs. In the intestine, mesenchymal cells play a critical role in controlling stemness, proliferation, and differentiation of epithelial cells [44]. It is well known that different types of mesenchymal cells secrete signaling molecules that regulate these processes in specific regions of the intestine, thereby shaping the proliferative crypt base and upper differentiated compartments. Thus, interactions between tumor cells and these mesenchymal cells could be critical for both the promotion and suppression of CRCs. Importantly, these mesenchymal cells are present in the submucosa but not outside or within the muscular layer. Therefore, to replicate the interactions between tumor cells and mesenchymal cells, it is crucial to transplant CRC cells into the epithelium or the underlying submucosal layer. In this regard, the CRC model developed in this study provides a more pathologically relevant microenvironment replicating interactions between tumors and mesenchymal cells from the initiation to the progression of tumors. Following engraftment, CRC cells are located in close proximity to mesenchymal cells present in the submucosa (Fig. 2), and they continuously recruit these mesenchymal cells throughout tumor progression (Figs. 4, 5). The presence of mesenchymal cells appears to be essential for the vigorous proliferation of CRC cells, as evidenced by the significant suppression of CRC cell proliferation observed in the central regions of larger tumors (Fig. 5E, F), where mesenchymal cells had not yet been recruited. Notably, the expression of the intestinal stem cell marker, ATAD2, is maintained in these quiescent regions (Fig. 6A), indicating that Caco-2 CRC cells can preserve their stemness independently of their proliferation status. The existence of quiescent CRC cells with high levels of stem cell marker expression within tumors has important pathological implications, as these cells may not be effectively targeted by conventional chemotherapy, which primarily affects proliferating cells. Consequently, our orthotopic CRC model successfully recapitulates significant tumor heterogeneity arising from variations in the microenvironment, which could affect therapeutic responses. The establishment of such a CRC model would be instrumental in assessing the efficacy of potential anticancer drugs and in the development of novel therapeutic strategies aimed at targeting quiescent CRC stem cells.

In our orthotopic CRC model, another important cell–cell interaction was observed between CRC cells and adjacent normal host epithelial cells. At the early stage of tumor development (one month after transplantation), the transplanted CRC cells were present directly adjacent to normal epithelial cells (Figs. 3, 4). Notably, the majority of the normal epithelial cells neighboring the transplanted CRC cells had already been quiescent and differentiated (Fig. 4). This observation aligns with recent studies that, in a genetic mouse model of intestinal adenomas, *Apc*-mutant intestinal cells act as supercompetitors against surrounding wild-type cells by secreting WNT antagonists during tumor development [15, 16]. Thus, it is plausible that human CRC cells harboring *APC* mutations might suppress the proliferation of adjacent normal epithelial cells through analogous mechanisms to achieve clonal expansion. In support of this notion, normal epithelial cells were absent in more advanced, larger tumors in this study (Fig. 5), suggesting that they had already been eradicated. The interaction between CRC cells and normal epithelial cells is not recapitulated in the previous CRC models, in which CRC cells are inoculated into the outside or within the muscularis externa, but not into the epithelial layer [8–12]. The orthotopic CRC model established in this study effectively replicates the competition between CRC cells and surrounding normal epithelial cells during the early stages of tumorigenesis, providing a valuable model for investigating the mechanisms by which CRC cells outcompete normal epithelial cells.

Metastasis represents the hallmark of cancer and is responsible for most cancer-related death. Therefore, reproducing CRC metastasis is one of the important applications of our orthotopic transplantation approach. Unfortunately, we could not address this issue with Caco2 cells, as metastasis was not observed in distant organs, including the liver, even six months after transplantation of Caco2 cells, despite the formation of large tumors (Supplementary Fig. S7). Therefore, it would be the next important issue in future research to replicate CRC metastasis using cancer cells with higher metastatic potential.

It should be noted that Caco-2 cells highly express TACSTD2, a fetal intestinal marker whose expression is often reactivated during the reprogramming of intestinal epithelial cells in tissue regeneration and CRC development [32, 33, 35] (Fig. 6B). The reprogramming of intestinal cells into the fetal intestine-like state has significant implications for CRC pathogenesis, as it is constitutively activated during colorectal tumorigenesis and is associated with drug resistance, metastasis and poor prognosis in CRC patients [32, 33, 35, 36]. Importantly, the expression of TACSTD2 in Caco-2 cells was not affected by the cellular location within the tumors, the presence or absence of mesenchymal cells, or the cell proliferation status (Fig. 6C). These findings suggest that the fetal intestinal signature can be robustly preserved in Caco-2 cells, irrespective of the surrounding tumor microenvironment. The clinicopathological relevance of fetal intestinal signatures in CRC has garnered increasing attention in recent years [34]. Our orthotopic Caco-2 xenograft model may serve as a valuable tool for investigating the significance of fetal intestinal signatures in CRC.

## Conclusion

In conclusion, this study presents a novel approach to establish an orthotopic model of CRC within the mouse cecum. In this approach, the entire transplantation process is visualized by externalizing the luminal surface of the cecum and directly accessing it under the guidance of a fluorescence stereoscopic microscope, thereby achieving efficient and reproducible engraftment of CRC cells into the cecal epithelium. Following transplantation, Caco-2 CRC cells initiate tumorigenesis within the natural tumor microenvironment, effectively mimicking the interactions between CRC cells and surrounding normal epithelial and mesenchymal cells. The development and progression of Caco-2 cell tumors in this model include sequential morphological changes from flat monolayer tumors to ACF-like tumors, protruding tumors and then large invasive tumors, highlighting the significance of tumor-microenvironment interactions in the CRC pathogenesis. The establishment of this orthotopic CRC model, which induces tumor development within a more natural microenvironment, should offer new opportunities to study the molecular mechanisms underlying CRC and to evaluate novel anticancer therapies in pathologically relevant contexts.

## Supplementary Information

Supplementary Material 1.

## Data Availability

The dataset and materials used in this study are available from the corresponding author upon reasonable request.

## Abbreviations

CRC: Colorectal cancer
DMEM: Dulbecco's modified Eagle’s medium
FBS: Fetal bovine serum
PBS: Phosphate-buffered saline
TBS: Tris-buffered saline
BSA: Bovine serum albumin
ACF: Aberrant crypt foci
EdU: 5-Ethynyl-2’-deoxyuridine
