# Supplementary material for: Establishment of a novel mouse model of colorectal cancer by orthotopic transplantation
Source: BMC Cancer. 2025 Mar 6;25:405. doi: 10.1186/s12885-025-13834-5 (PMC11884030; doi:10.1186/s12885-025-13834-5)
Supplement: Supplementary file 1 — Supplementary Material 1. [file 12885_2025_13834_MOESM1_ESM.pdf]

Supplementary Material

Establishment of a novel mouse model of colorectal cancer by orthotopic transplantation

Cewen Chen[1,2], Qiaochu Fu[1,2], Lei Wang[1,2], Shinya Tanaka[1,2], Masamichi Imajo[2*]

[1]Department of Cancer Pathology, Faculty of Medicine, Hokkaido University, N15, W7, Kita-ku, Sapporo 060-8638, Japan
[2]Institute for Chemical Reaction Design and Discovery (WPI-ICReDD), Hokkaido University, N21, W10, Kita-ku, Sapporo 001-0021, Japan

[*]To whom correspondence should be addressed. E-mail: m-imajo@icredd.hokudai.ac.jp

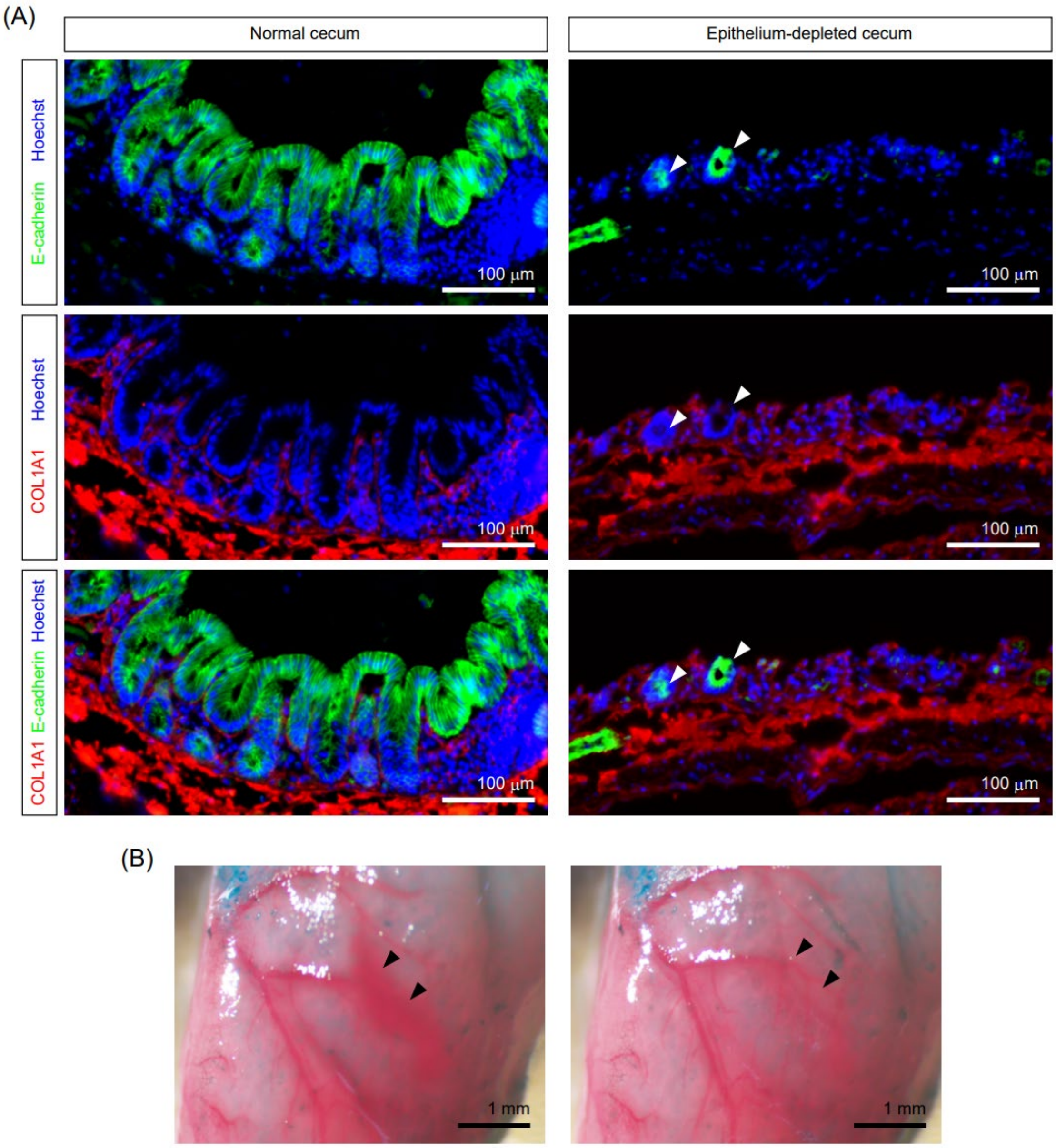

Supplementary Fig. S1 Removal of the cecal epithelium by mechanical brushing. **A** Immunofluorescence analysis of the cecum immediately after epithelial removal. Most, but not all, E-cadherin-positive epithelial cells were successfully removed by brushing. Remaining epithelial cells are indicated by white arrowheads. **B** Minor bleeding was occasionally observed during the epithelial removal (left, black arrowheads), which could be halted by applying pressure with a cotton swab for few minutes (right).

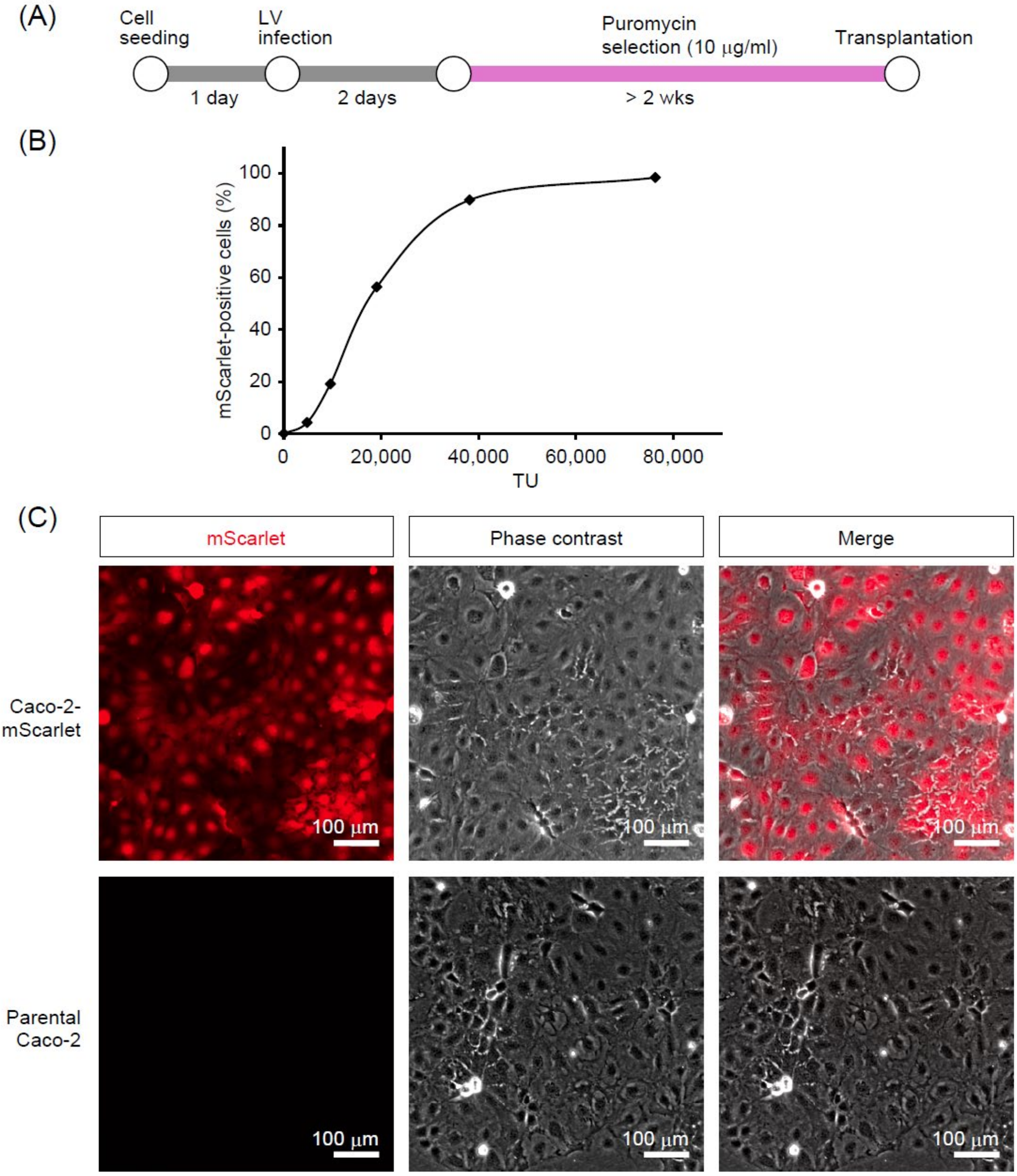

Supplementary Fig. S2 Generation of Caco-2 cells stably expressing mScarlet. **A** Experimental timeline for establishing Caco-2 cells stably expressing mScarlet. Cells were infected with a lentivirus simultaneously expressing mScarlet and the puromycin resistance gene (*mScarlet-IRES-puro*) one day after seeding. Two days after infection, maScarlet-expressing cells were selected with puromycin (10 μg/ml). The selection was continued for more than two weeks until the cells were used for transplantation. **B** The percentage of mScarlet-expressing Caco-2 cells two days after infection. The infection efficiency increased as the lentiviral titer

(transduction unit (TU)) increased. Approximately 90% of cells were infected with the viral solution at 40,000 TU. **C** Images showing mScarlet-expressing and parental Caco-2 cells. After two weeks of selection, puromycin was removed from the culture medium. Stable expression of mScarlet was maintained for two weeks after the removal of puromycin.

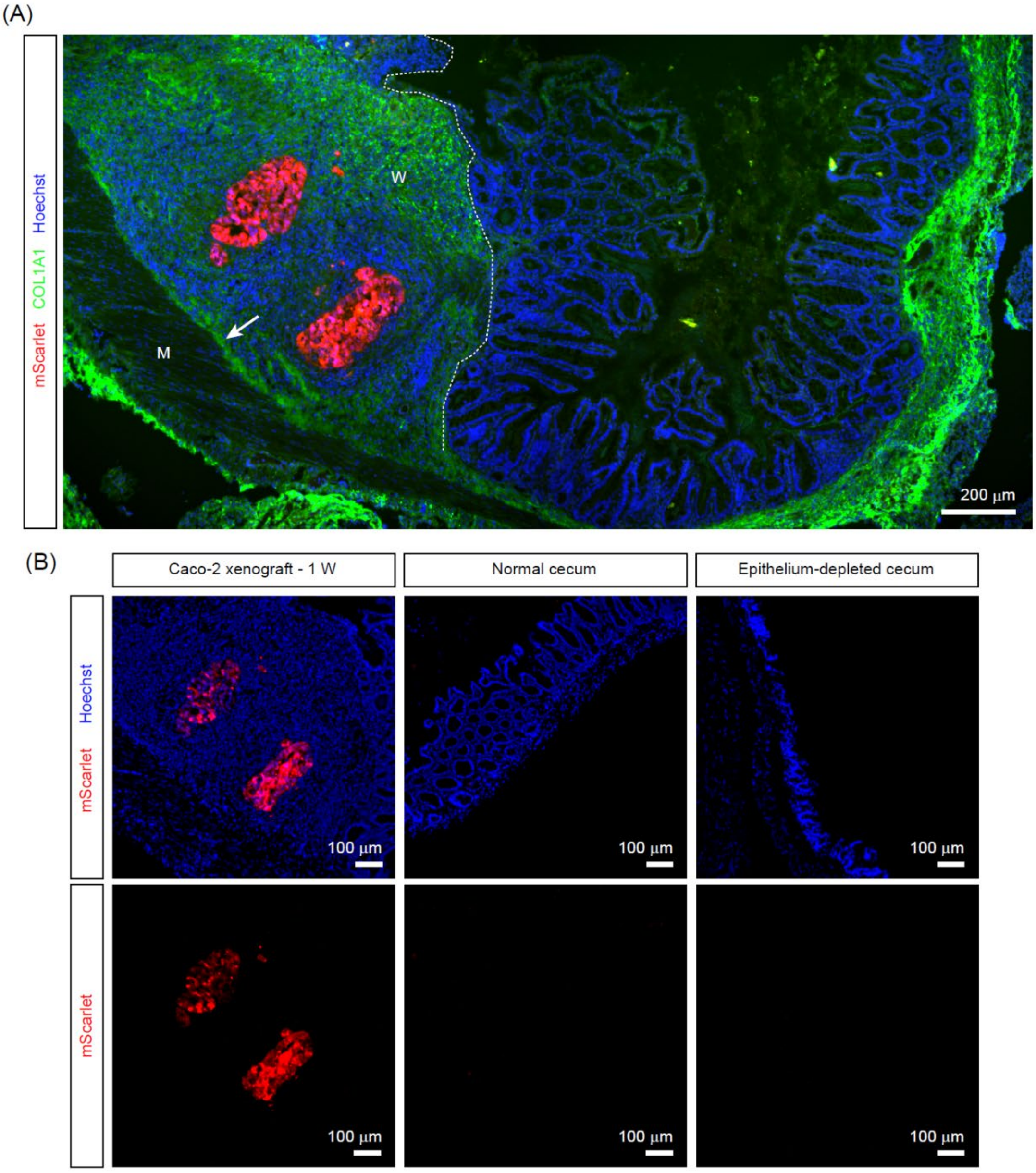

Supplementary Fig. S3 Analysis of Caco-2 cell engraftment to the cecum. **A** Formation of wound beds one week after transplantation. One week after transplantation, wound beds (W) containing many stromal cells developed in the epithelium-depleted region. Caco-2 cells seeded on the cecum surface were overlaid by a wound bed. The white arrow marks the boundary between the muscular (M) and submucosal layers. **B** Detection of mScarlet fluorescence in the cecal sections. mScarlet fluorescence was detected in the cecum transplanted with Caco-2-mScarlet cells one week after transplantation (left), but not in the untransplanted normal tissues (middle) or epithelium-depleted tissues (right).

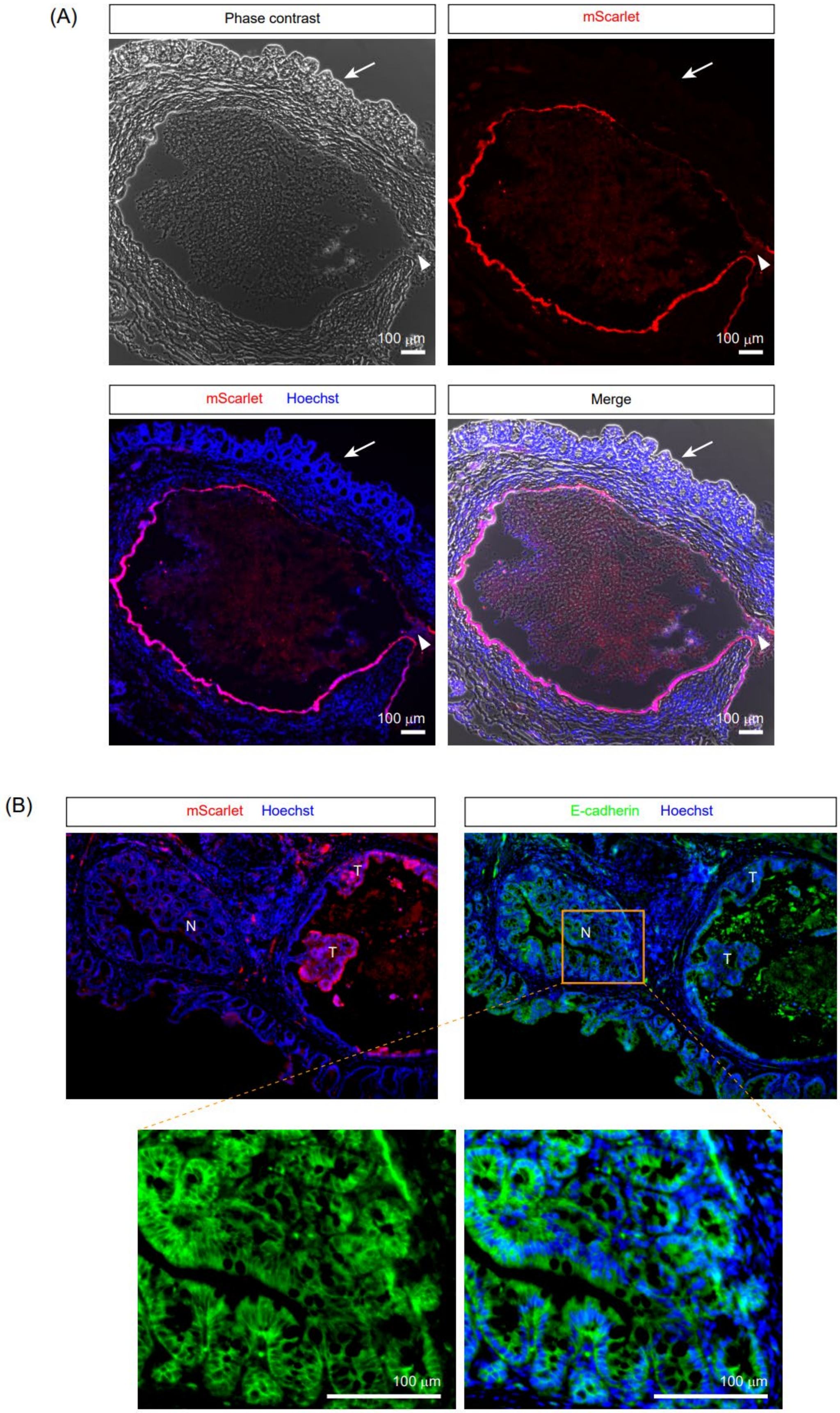

(A)
Phase contrast
mScarlet
mScarlet Hoechst
Merge
100 µm
(B)
mScarlet Hoechst
E-cadherin Hoechst
N
T
100 µm

Supplementary Fig. S4 Analysis of tumors one month after transplantation. **A** Phase contrast and fluorescence images of the cecum one month after transplantation. A tumor with large central lumen was observed. The tumor lumen was connected to the intestinal lumen (white arrowheads). The region containing the tumor was covered by the normal epithelium (white arrows). **B** One month after transplantation, large cellular masses comprising mScarlet-negative and E-cadherin-positive normal epithelial cells (N) as well as Caco-2 tumors (T) were occasionally observed.

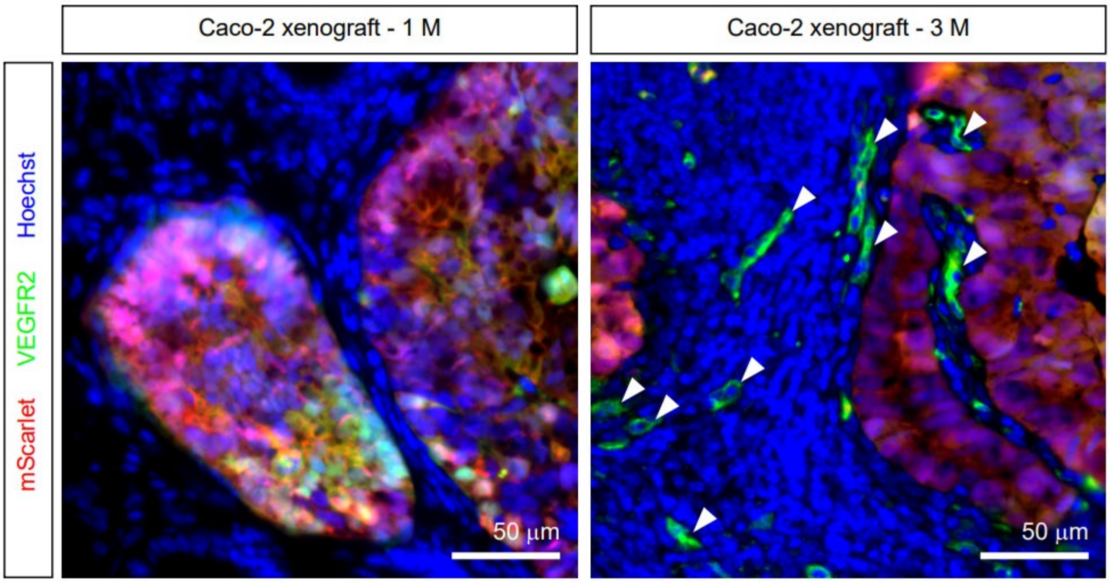

Supplementary Fig. S5 Analysis of tumor angiogenesis by immunofluorescence of VEGFR2. One month after transplantation, VEGFR2-positive endothelial cells were scarce around the Caco-2 cell tumors (left). However, three months after transplantation, VEGFR2-positive endothelial cells were abundant around the tumors (white arrowheads). Caco-2 cells also showed VEGFR2 expression.

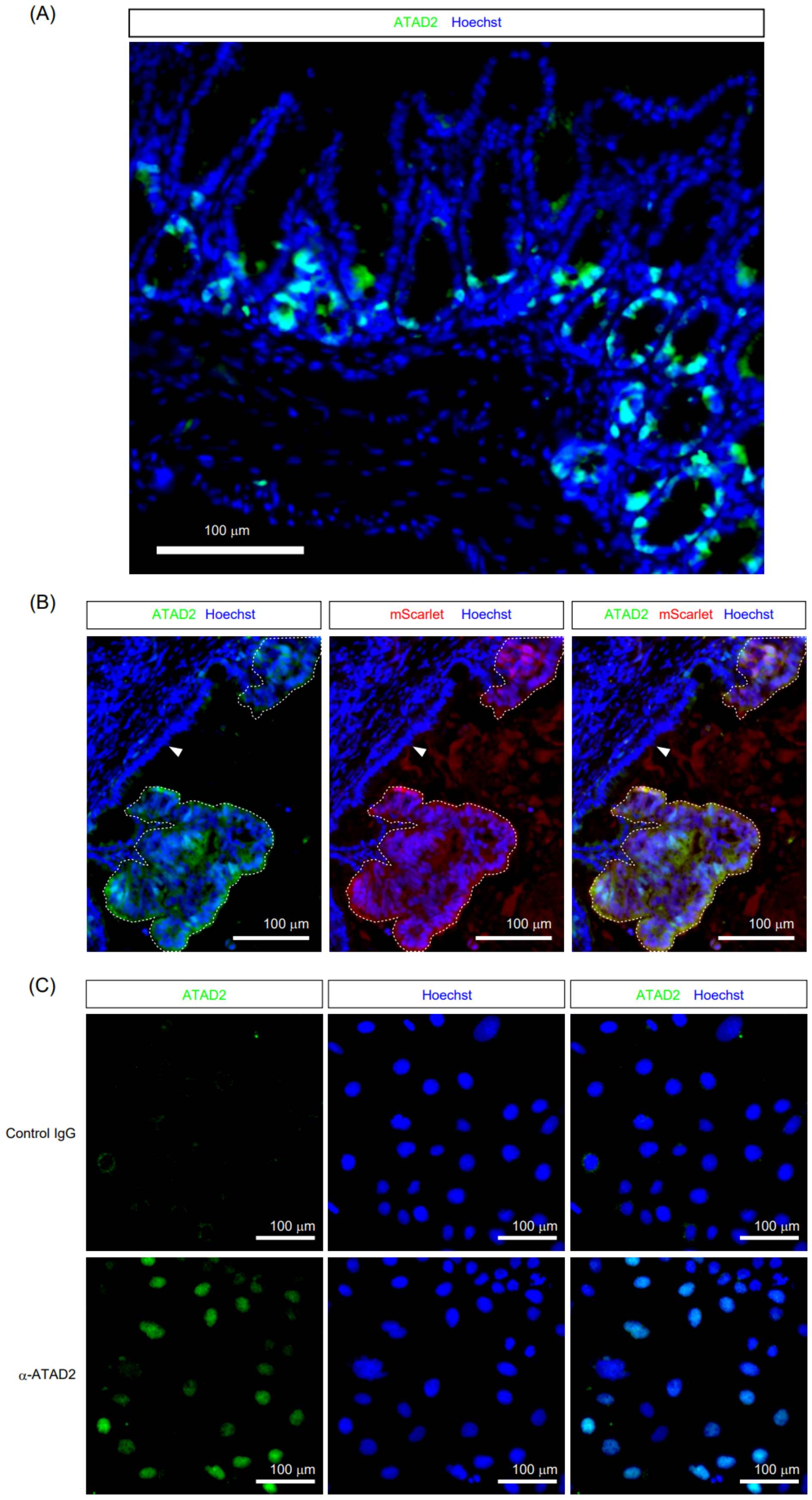
(A)
ATAD2 Hoechst
100 μm
(B)
ATAD2 Hoechst
mScarlet Hoechst
ATAD2 mScarlet Hoechst
100 μm
100 μm
100 μm
(C)
ATAD2
Hoechst
ATAD2 Hoechst
Control IgG
α-ATAD2
100 μm

Supplementary Fig. S6 Caco-2 cells maintain high ATAD2 expression in both *in vitro* culture and *in vivo* tumor environments. **A-C** Immunofluorescence analysis of ATAD2. **A** In the normal cecum epithelium, ATAD2-positive cells were present in the crypt bottom region. **B** In early-stage Caco-2 cell tumors, ATAD2 was ubiquitously expressed in most tumor cells, while normal epithelial cells adjacent to tumors were negative for ATAD2 (white arrowheads). **C** ATAD2 was expressed in Caco-2 cells cultured under standard conditions.

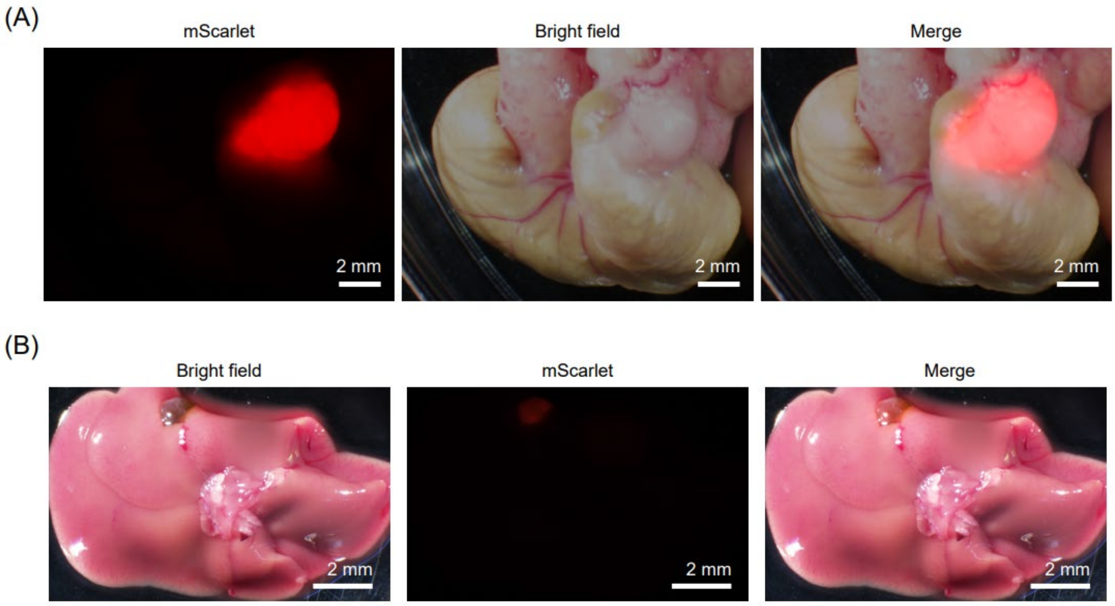

Supplementary Fig. S7 Gross view of the cecum and liver six month after transplantation of Caco-2-mScarlet cells. **A** Large tumor foci expressing mScarlet were visible from the outside of the cecum. **B** No metastasis of Caco-2 cells was observed in the liver.
